# Evaluation of serum ATX and LPA as potential diagnostic biomarkers in patients with pancreatic cancer

**DOI:** 10.1186/s12876-021-01635-6

**Published:** 2021-02-10

**Authors:** Jiang Chen, Hongyu Li, Wenda Xu, Xiaozhong Guo

**Affiliations:** Department of Gastroenterology, General Hospital of Northern Theater Command, No. 83 Wenhua Road, Shenyang, 110840 Liaoning Province China

**Keywords:** Biological markers, Diagnosis, Lysophospholipids, Autotaxin, Pancreatic neoplasms

## Abstract

**Background:**

Pancreatic cancer (PC) is a devastating disease that has a poor prognosis and a total 5-year survival rate of around 5%. The poor prognosis of PC is due in part to a lack of suitable biomarkers that can allow early diagnosis. The lysophospholipase autotaxin (ATX) and its product lysophosphatidic acid (LPA) play an essential role in disease progression in PC patients and are associated with increased morbidity in several types of cancer. In this study, we evaluated both the potential role of serum LPA and ATX as diagnostic markers in PC and their prognostic value for PC either alone or in combination with CA19-9.

**Methods:**

ATX, LPA and CA19-9 levels were evaluated using ELISA of serum obtained from PC patients (n = 114) healthy volunteers (HVs: n = 120) and patients with benign pancreatic diseases (BPDs: n = 94).

**Results:**

Serum levels of ATX, LPA and CA19-9 in PC patients were substantially higher than that for BPD patients or HVs (*p* < 0.001). The sensitivity of LPA in early phase PC was 91.74% and the specificity of ATX was 80%. The levels of ATX, LPA and CA19-9 were all substantially higher for early stage PC patients compared to levels in serum from BPD patients and HVs. The diagnostic efficacy of CA19-9 for PC was significantly enhanced by the addition of ATX and LPA (*p* = 0.0012).

**Conclusion:**

Measurement of LPA and ATX levels together with CA19-9 levels can be used for early detection of PC and diagnosis of PC in general.

## Background

Early diagnosis and improved treatments have together contributed to increased survival rates for several types of cancer over the recent decades. However, pancreatic cancer (PC) continues to be one of the deadliest cancers and its prognosis remains poor [1]. In patients with resect-able tumors, the 5-year survival rate for PC ranges from 15 to 20%. For those PC patients presenting with unresectable tumors and distant metastases, the 5-year survival rate is nearly 0% [2]. This poor prognosis and low survival rate are largely due to a lack of recognizable symptoms and early detection markers that result in delays in diagnosis [3]. Thus, there is an urgent need for new biomarkers that can allow early detection of PC and reduce the likelihood of metastasis.

CA19-9 is regarded as a gold standard marker that is applied for diagnosis (screening and staging) and monitoring of PC progression (measure of resectability, early identification of recurrence, and prediction of therapeutic response) [4]. However, as a biomarker, CA19-9 has several limitations for diagnosing early stage PC including low expression levels and specificity as well as inconsistent presentation in PC patients [5]. Therefore, new, more sensitive biomarkers that can be used either alone or in combination with previously defined biomarkers are needed to increase the likelihood of early PC detection [6].

Increasing evidence now supports a physiological role for lysophosphatidic acid (LPA) in regulating tumor progression, angiogenesis, and metastasis. LPA is a bioactive phospholipid that engages at least six receptors, LPAR1-6, which are each coupled to a distinct G-protein [7] that participates in various cellular activities such as cell migration, proliferation, and differentiation [8]. LPA also contributes to progression, metastasis, and initiation of various cancers, including PC [9]. LPA is present in various biological fluids, and its levels in plasma are well-characterized in terms of its role in blood coagulation. LPA is generated from lysophospholipase D (lysoPLD)-mediated hydrolysis of lysophosphatidylcholine (LPC) [10]. Total plasma LPA is considered to be a biomarker for diagnosis of gastric tumors [11].

Autotaxin (ATX), also referred to as ectonucleotide pyrophosphatase / phosphodiesterase 2 (ENPP2), is a 125 kDa secreted glycoprotein that was originally discovered as an autocrine cell motility factor produced by melanoma cells cultured in conditioned medium [12]. ATX was primarily thought to act as a pyrophosphatase [13], but subsequent studies showed that ATX could hydrolyze LPC to form LPA [14]. Augmented ATX expression has been reported in breast cancer, PC, hematological cancer, and thyroid and hepatocellular carcinoma [15]. ATX levels can be correlated with cancer cell invasiveness [16, 17].

Ex vivo and in vivo studies showed that elevated ATX-LPA signaling activity can contribute to cancer progression and initiation [18].]. However, whether plasma levels of ATX and LPA could serve as diagnostic biomarkers for PC is unclear. In this study, we evaluated the potential of plasma LPA and ATX as cancer biomarkers and assessed whether they could be used alone or in combination with CA19-9 to allow early detection of PC.

## Methods

### Patients

Serum samples were gathered from 328 individuals, who were divided into three groups: Group A had 114 PC patients, Groups B and Group C served as cohort control groups and comprised 120 healthy volunteers (HVs) and 94 patients with benign pancreatic diseases (BPDs), respectively. All the patients were consecutively admitted to the General Hospital of Northern Theater Command, Shenyang, China between January 2013 and May 2019. The research protocol was adopted through the Ethics Committee of the General Hospital of Northern Theater Command, and the number was K(2012)49. Informed written consents were obtained for all participants.

"High sugar diet" was defined according to the standard of the American Heart Association (AHA) [19]. For men, Eating more than 37.5 g or 9 teaspoons of sugar a day is considered a high sugar diet. For women, the standard was changed to 25 g or 6 teaspoons of sugar per day. The "Alcohol consumption" was defined according to the standard of the National Institute on Alcohol Abuse and Alcoholism (NIAAA) [20]. The excessive drinking is 14 × 8 g pure alcohol / day or 14 × 20 g pure alcohol / week for men, and 14 × 4 g pure alcohol / day or 14 × 8 g pure alcohol / week for women. In this study, High sugar diet and alcohol consumption were ascertained at all visits by means of an interviewer-administered dietary questionnaire. In calculating the amount of sugar or alcohol consumed (in grams per day or per week), people were judged whether they currently with the high sugar diet or excessive drinking.

CT scan and chest X-ray, as well as biological and physical examinations, were conducted during pre-treatment investigations. All patients in all groups had regular blood chemistry and full clinical tests. Imaging, blood chemistry and physical test results for the HVs were within normal ranges. Patients with PC were diagnosed based on histopathological or clinical observations (history of pancreatic disease, clinical presentation, laboratory tests and abdominal imaging). None had undergone radio- or chemotherapy prior to serum collection. Diagnosis of BPDs was based on the absence of malignant features on imaging studies for a one year follow-up.

### Samples

A total of 10 mL of venous blood was collected from each participant into sterile vacutainers. The samples were centrifuged at 3,500 g for 10 min and serum aliquots were stored at -80 ˚C. The samples were thawed prior to measurement of ATX, LPA and CA19-9 blood levels. Blood biochemical parameters including total bilirubin, albumin, alanine alkaline phosphatase, aspartate aminotransferase, γ-glutamyl transferase, transaminase, glucose, creatinine, prothrombin time, D-dimer and activated partial prothrombin time were measured using a Siemens ADVIA1800 chemistry analyzer.

Serum levels of LPA, ATX and CA19-9 were assayed by ELISA (Cloud-Clone Corp., TX, USA) and estimated by interpolation from a standard curve produced using known amounts of each protein. The samples were serially diluted with PBS and assessed again to ensure that the concentrations were within measurable ranges. The investigators were blinded to sample origin and all tests were executed in triplicate following the manufacturer’s instructions.

### Statistical analysis

All statistical analyses were executed using the SPSS v20.0 package. Uninterrupted data are shown as median with range and mean ± standard error of the mean (SEM). An independent sample *t-*test was used to compared continuous data. Differences among categories were examined using the Mann–Whitney U test. To describe the prognostic precision performance of the three markers as diagnostic biomarkers, receiver operating characteristics (ROC) analysis was used. The AUC was calculated and compared by the De Long test. Reversible *p* < 0.05 was regarded as statistically significant.

## Results

### Characteristics of PC and BPD patients and healthy volunteers

Overall, 120 HVs, 94 patients with BPDs and 114 PC patients were enrolled in this study. The patient demographics are summarized in Table 1. Of the 114 PC patients, 36 (31.6%), and 78 (68.4%) were female and male, respectively. The age of this group ranged from 40 to 82 years-old, and the median age was 59 years-old. The median tumor size was 3.94 cm (range from 1.0–6.2 cm). For the majority of PC patients (81 cases, 71.1%), the tumor was located in the head of the pancreas, followed by the tail or body (33 cases, 28.9%). Regional lymph node metastasis (LNM) was present in 14 (36.8%) PC patients, absent in 18 patients (47.3%) and 2 patients (5.2%) had duodenum metastasis. Pancreatic ductal adenocarcinoma was diagnosed for 107 patients whereas 7 patients had pancreatic acinar cell carcinoma. Pathologic grade I was seen for 12 patients (10.5%), whereas grade II and grade I to II was seen for 84 (73.7%) and 18 (15.8%) patients, respectively. Perineural invasion (PNI) was observed for 109 cases (95.6%). In terms of tumor stage, 9 (7.7%), 35 (31.6%), 18 (15.8%), and 51 (44.9%) cases were Stage 1, 2, 3, and 4, respectively. Autoimmune pancreatitis (n = 8, 5 males and 3 females), chronic pancreatitis (n = 62, 51 males and 11 females) and pancreatic cyst disease (n = 24, 17 males and 7 females) were represented among the 94 BPD patients.

**Table 1:** Characteristics of patients and healthy volunteers

Characteristics	PC patients (n = 114)		Patients with BPDs (n = 94)		HVs (n = 120)		*Χ*^2^	*p* value
	No	%	No	%	No	%		
Age (years)								
Median (range)	59 (40–82)		56 (31–83)		60 (37–84)			0.642
Sex								
Female	36	31.6	27	28.7	36	30	6.183	0.792
Male	78	68.4	67	71.3	84	70	0.169	0.572
High sugar diet	54	47.3	15	16.1	24	20	4.097	0.043*
Smoker	60	52.6	11	11.7	26	21.7	5.653	0.017*
Alcohol consumption	6	5.26	5	5.3	6	5	0.002	0.966
Family history of PC	15	13.2	7	7.4	6	5	0.924	0.336
Location								
Head	81	71.1						
Body/rear	33	28.9						
Histologic grade								
I	12	10.5						
II	84	73.7						
I–II	18	15.8						
Tumor size (cm)								
Median	3.94							
Range	1–6							
LNM								
Positive	42	36.8						
Negative	54	47.3						
Unknown	18	15.9						
PNI								
Positive	109	95.6						
Negative	5	4.4						
Tumor stages (TS)								
1b	9	7.8						
2a	21	18.4						
2b	15	13.1						
3	18	15.8						
4	51	44.9						

BPDs, benign pancreatic diseases; LNM, lymph node metastasis; PNI, perineural invasion; HVs, healthy volunteers

^*^Depicts results were significant at *p* < 0.05

Compared with the cohort control groups, the PC patient group had a significantly higher proportion of smokers and those who consumed a diet high in sugar (*p* < 0.05). Age, sex, presence of chronic pancreatitis, alcohol consumption, and family history of PC were statistically similar among all three groups.

### Biochemical parameters for healthy volunteers, PC patients, and BPD patients

Results for analysis of blood samples obtained from 120 HVs and 94 BPD patients were analyzed by comparison with 114 PC patients. The PC patients had not undergone radio- or chemotherapy prior to the blood collection. PC patients had alanine transaminase, aspartate aminotransferase, alkaline phosphatase, and γ-glutamyl transferase activity that was 2.3-, 2.8-, 1.5-, and 2.3-fold higher than that for the HVs (*p* < 0.05). Meanwhile, the total serum level of bilirubin, creatinine, and D-dimer content in PC patients was 6.2-, 1.3-, and 3.8-fold higher than that for HVs (*p* < 0.001). Compared with the HVs, the blood glucose level of PC patients was 1.8-fold higher (*p* < 0.05) and the activated partial prothrombin time expressed as prothrombin time was 1.4-fold longer (*p* < 0.05). The albumin content in PC patients was lower (*p* < 0.05) compared with that in HVs (Table 2).

**Table 2:** Comparison of biochemical parameters among PC patients, patients with benign pancreatic diseases and healthy volunteers

Variables	PC patients (n = 114)	Patients with BPDs (n = 94)	HVs (n = 120)	*p* value (s)	
	Median (range)	Median (range)	Median (range)	PC *vs.* BPDs	PC *vs.* HVs
Total bilirubin, µmol/L	44.8 (19.2–141.5)	9.16 (6.3–72.8)	7.24 (3.68–15.1)	< 0.001	< 0.001
Albumin, g/L	31.48 (20.1–83.5)	42 (39.7–65.4)	51.3 (27.1–168.3)	< 0.001	< 0.001
Alanine transaminase, U/L	42 (34.1–144)	24.3 (24.3–59.5)	18.8 (13.3–41)	< 0.001	< 0.001
Aspartate aminotransferase, U/L	116.9 (62–328.4)	37.5 (27.9–212.4)	27.2 (18–59.6)	< 0.001	< 0.001
Alkaline phosphatase, U/L	109.7 (39.2–247.6)	80.4 (19.3–169.2)	71.2 (41.5–145.2)	0.008*	0.005*
γ-glutamyl transferase, U/L	102.3 (77.5–201.2)	51.4 (25–110.2)	44.7 (37.2–88.6)	0.005*	0.004*
Glucose, mmol/L	9.3 (8.8–28.7)	8.7 (3.4–42.6)	4.5 (4.1–8.2)	0.086	< 0.001
Creatinine, µmol/L	87.3 (45.3–143.3)	71.2 (19.3–95.5)	64.7 (32.6–213.8)	0.043*	0.027*
Prothrombin time, sec	17.3 (4.5–56.1)	18.4 (8.7–92.6)	12.6 (10.6–58)	0.074	0.018*
Activated partial prothrombin time, sec	44.5 (37.4–103.5)	27.3 (10.9–90.1)	30.5 (22.2–67.5)	< 0.001	< 0.001
D-dimer, µg/mL	0.8 (0.4–2.3)	0.3 (0.08–1.9)	0.2 (0.03–1.2)	< 0.001	< 0.001

BPDs, benign pancreatic diseases; HVs, healthy volunteers

^*^Depicts results were significant at *p* < 0.05

Comparing PC patients with BPD patients, PC patients had alanine transaminase, aspartate aminotransferase, alkaline phosphatase, and γ-glutamyl transferase activities that were increased by 1.75-, 2.05-, 1.36-, and 1.9-fold (*p* < 0.05) more than that for BPD patients. The total content of bilirubin, creatinine, and D-dimer contents in PC patients was 4.9-, 1.2-, and 2.6-fold higher than that for BPD patients (*p* < 0.001). PC and BPD patients had similar blood glucose levels and activated partial prothrombin time (*p* > 0.05). The albumin content in PC patients was depressed (*p* < 0.001) compared to that for BPD patients (Table 2).

### Comparison of ATX, LPA, and CA19-9 serum concentrations for HVs, PC patients, and BPD patients

The plasma levels of ATX, LPA and CA19-9 in PC patients were significantly higher than that for the HV group (*p* < 0.001) and the BPD group ( *p* < 0.001) (Table 3). Upon considering tumor stage, ATX, LPA and CA19-9 levels for TS1 & TS2 patients were all significantly higher (*p* = 0.023, *p* = 0.044 and *p* < 0.001, respectively) than those for the BPD group. Meanwhile, both ATX and CA19-9 in TS1 & TS2 were significantly higher (*p* = 0.005 and *p* < 0.001, respectively) compared to the HV group. However, LPA levels between the TS1 & TS2 group and HVs did not differ significantly (*p* = 0.057). When the three biomarkers were segregated according to tumor stage, a significant elevation was seen for all three markers in patients with later PC stage (TS 3 & TS4) compared to those with early PC stage (TS 1 & TS2) (*p* < 0.001).

**Table 3:** Comparison of the concentrations of biochemical parameters among healthy volunteers, patients with PC and benign pancreatic diseases

Biomarkers	HVs	BPDs	PCs			*p* value (s)				
	(n = 120)	(n = 94)	Total (n = 114)	TS1 + TS2 (n = 45)	TS3 + TS4 (n = 69)	PC vs. HVs	PC vs. BPDs	TS1 + TS2 vs. HVs	TS1 + TS2 vs. BPDs	TS1 + TS2 vs. TS3 + TS4
ATX (ng/mL)										
Median	255.3	267.5	392.6	294.9	422	< 0.001	< 0.001	0.005*	0.023*	< 0.001
Range	130–1408	184–977	297–1753	262–1455	312–1620					
LPA(ug/mL)										
Median	10.8	9.83	17.48	15.75	24.6	0.004*	< 0.001	0.057	0.044*	0.002*
Range	5.2–49	4.9–28.2	8.1–97	8.3–92	9.5–97.3					
CA19-9 (U/mL)										
Median	36.3	51.2	185.4	131.1	217.4	< 0.001	< 0.001	< 0.001	< 0.001	< 0.001
Range	13–69.1	21–105.3	24–970	23–289	32–970					

HVs, healthy volunteers; PC, pancreatic cancer; TS, tumor stage

^*^Depicts significance at *p* value < 0.05

### Diagnostic evaluation of serum ATX and LPA in PC patients using CA19-9 as a standard marker

**Fig. 1:**
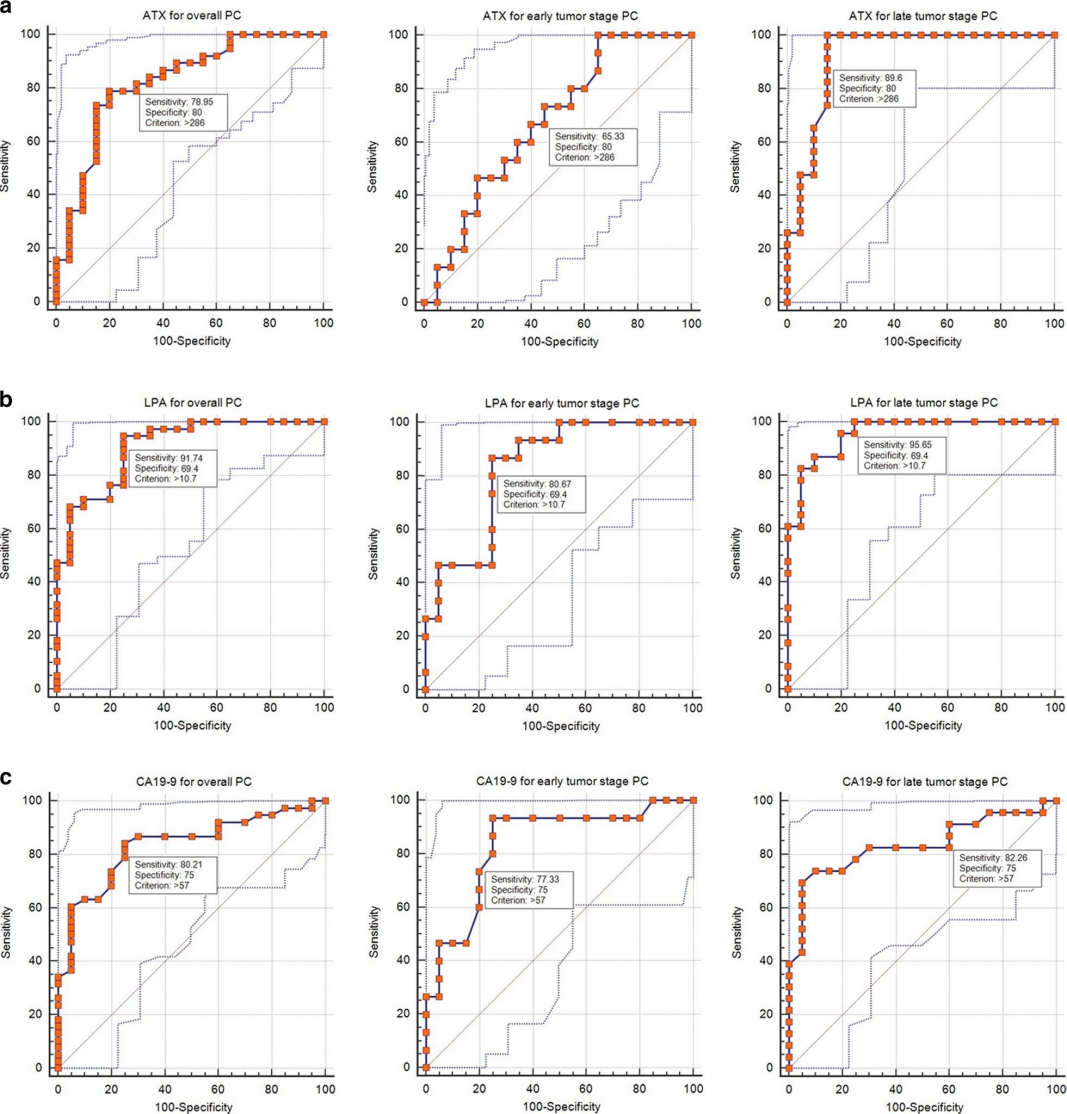
Receiver Operating Characteristic (ROC) curve analysis of serum ATX, LPA, and CA19-9 levels for prediction of overall, early tumor stage, and late tumor stage PC relative to levels in BPD patients and HVs

The specificity and sensitivity of serum ATX and LPA levels were next assessed for PC diagnosis using CA19-9 as a standard diagnostic marker. The diagnostic efficacy of the three markers was assessed at 286 ng/mL, 10.7 µg/mL and 57 U/mL, which were selected as cut-off values for ATX, LPA and CA19-9, respectively (Fig. 1, Table 4). LPA had the highest sensitivity among all PC patients (91.74%), and these values varied for late tumor stage (95.65%) and early tumor stage (85.67%) patients. Sensitivity was lower for ATX and CA19-9. CA19-9 had a sensitivity of 80.21% among all PC patients, and the sensitivity was 82.26% and 77.33% for late and early tumor stage, respectively. Meanwhile, ATX had a sensitivity of 78.95% for all PC patients, and for early and late tumor stage the values were 65.33% and 89.6%, respectively. When tumor stage was disregarded, ATX was the most specific biomarker (80%) followed by CA19-9 (75%) and LPA (69.4%) compared to healthy volunteers.

**Table 4:** Sensitivity and specificity of potential diagnostic biomarkers in pancreatic cancer

Biomarkers	Optimum cut-off	Sensitivity (%)	Specifificity (%)
PC vs. Controls			
CA19-9	≥ 57 U/mL	80.21	75
ATX	≥ 286 ng/mL	78.95	80
LPA	≥ 10.7 µg/mL	91.74	69.4
TS1 + TS2 vs. Controls			
CA19-9	≥ 57 U/mL	77.33	75
ATX	≥ 286 ng/mL	65.33	80
LPA	≥ 10.7 µg/mL	80.67	69.4
TS3 + TS4 vs. Controls			
CA19-9	≥ 57 U/mL	82.26	75
ATX	≥ 286 ng/mL	89.6	80
LPA	≥ 10.7 µg/mL	95.65	69.4

Controls: healthy volunteers + benign pancreatic diseases

TS, tumor stage

### Combining ATX and LPA with CA19-9 enhanced the diagnostic precision for early tumor stage PC

**Fig. 2:**
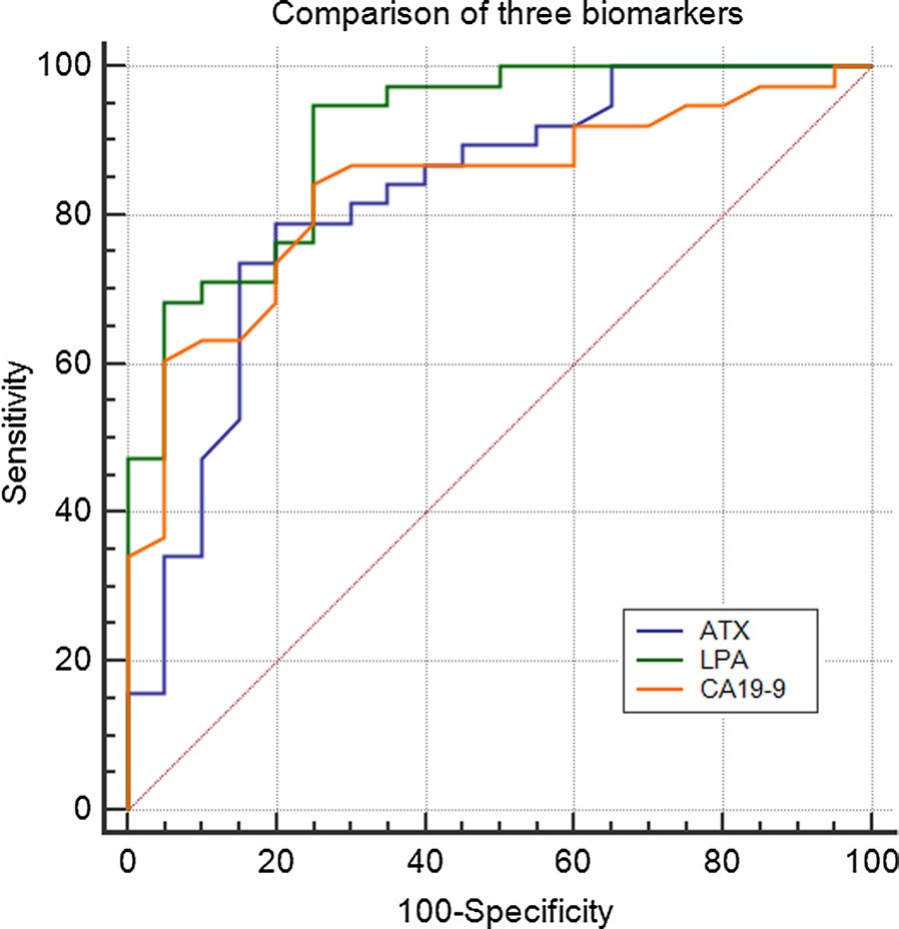
Receiver Operating Characteristic (ROC) curve comparing ATX, LPA, and CA19-9 levels for early detection of PC relative to levels in BPD patients and HVs. The Areas Under the ROC Curve (AUROC) were 0.836 for CA19-9, 0.824, for ATX, and 0.908 for LPA

We next attempted to determine whether combining ATX and LPA with CA19-9 improved the diagnostic accuracy for early stage PC. Compared with the HV group, the combination of ATX with CA19-9 enhanced the Areas Under the ROC Curves (AUROC) significantly (*p* = 0.0268), whereas adding the three biomarkers together further improved AUROC to 0.983 ± 0.016 (*p* = 0.0012). Furthermore, the combination of ATX with CA19-9 also enhanced the Areas Under the ROC Curve (AUROC) significantly (*p* = 0.0381) and adding all three biomarkers together further improved the AUROC to 0.973 ± 0.023 (*p* = 0.0090) when compared with the BPD group (Table 5 and Fig. 2). Upon combining two or all three markers, the AUROC increased for the late tumor stage of PC, but the increase was not significant (CA19-9 and ATX *vs.* HV group, *p* = 0.4508) (CA19-9 and ATX *vs.* BPD group, *p* = 0.279) (ATX, LPA and CA19-9 *vs.* HV group, *p* = 0.1657) ( ATX, LPA and CA19-9 *vs.* BPD group, *p* = 0.136) ( Table 5 and Fig. 2).

**Table 5:** Potential combined ATX and/or LPA with CA19-9 biomarkers

Biomarkers	AUROC ± SEM	95% CI	*p* value
TS1 + TS2 vs. HVs			
CA19-9	0.837 ± 0.0423	0.673–0.939	
CA19-9 + ATX	0.953 ± 0.0309	0.823–0.996	0.0268*
CA19-9 + ATX + LPA	0.983 ± 0.016	0.970–0.999	0.0012*
TS1 + TS2 vs. BPDs			
CA19-9	0.821 ± 0.065	0.758–0.972	
CA19-9 + ATX	0.926 ± 0.041	0.849–0.993	0.0381*
CA19-9 + ATX + LPA	0.973 ± 0.023	0.921–0.999	0.0090*
TS3 + TS4 vs. HVs			
CA19-9	0.836 ± 0.064	0.691–0.931	
CA19-9 + ATX	0.898 ± 0.0516	0.767–0.969	0.4508
CA19-9 + ATX + LPA	0.991 ± 0.0917	0.902–0.999	0.1657
TS3 + TS4 vs. BPDs			
CA19-9	0.835 ± 0.094	0.714–0.951	
CA19-9 + ATX	0.881 ± 0.066	0.827–0.992	0.279
CA19-9 + ATX + LPA	0.942 ± 0.078	0.913–0.999	0.136

HVs, healthy volunteers; BPDs, benign pancreatic diseases; TS, tumor stage; SEM, standard error of the mean; AUROC, Areas Under the ROC Curves

^*^Depicts significance at *p* value < 0.05

## Discussion

Early diagnosis of PC can markedly increase the probability of successful radical surgery as well as improve disease outcome and prolong survival time [21]. Novel biomarkers or a suite of biomarkers are needed to allow early detection of PC.

Recently, a set of new markers derived from serum was recently assessed as PC markers [5]. Among them, increasing attention is being paid to the ATX-LPA signaling axis, which regulates a number of cellular activities [15]. In particular, levels of LPA are related to activated platelets that can both directly and indirectly promote tumor growth, metastasis, and immune evasion [22]. Moreover, LPA is reported to induce cell proliferation, migration, and invasion of gastric tumors such as PC [11]. The extracellular lysoPLD ATX catalyzes the conversion of LPC to LPA, and overexpression of ATX results in multiple physiological abnormalities [23]. As for PC, Nakai et al. [24] have found that among patients with various cancers of digestive system, increased serum ATX activity was predominantly observed among pancreatic cancer patients. Meanwhile, overexpression of both ATX and LPA in pancreatic tissues has been reported for PC patients and the ATX-LPA axis played a critical role and might be the potential target in pancreatic cancer [25–27]. These findings suggest that measuring serum levels of ATX and LPA, either alone or in combination with the gold standard PC biomarker CA19-9 [4], could help distinguish patients with PC. In this study, we found that PC patients had significantly increased serum ATX and LPA levels, relative to HVs and BPD patients who had conditions such as benign biliary obstruction or chronic pancreatitis. The results also indicated that ATX and LPA levels may be affected by jaundice and the level of alkaline phosphatase. Furthermore, levels of all three biomarkers in samples from early tumor stage PC patients significantly differed from those seen for patients with late tumor stage. Together these results support the use of serum ATX and LPA as a diagnostic biomarker for PC.

An ideal tumor biomarker should have high sensitivity and high specificity. Biomarkers that have high sensitivity can detect related malignant tumors at an early stage, whereas biomarkers having high specificity are useful to make an accurate differential diagnosis [28]. In this study, CA19-9, when cut-off value was 57 U/mL, had 75% specificity and 80.21% sensitivity for the detection of PC among the entire study population, yet for detection of early PC (TS1 and 2) this sensitivity decreased to 77.33%. Meanwhile, LPA, when cut-off value was 10.7 µg/mL, had 91.74% sensitivity and 69.4% specificity for detection of PC from among the total study population. Considering early stage PC patients, the sensitivity was 80.67%. ATX, when cut-off value was 286 ng/mL, had 80% specificity for early stage PC detection, but the sensitivity was 65.33%. It was noteworthy that elevated serum ATX can be seen in tumors, autoimmune diseases, liver cirrhosis and other multiple system and organ diseases [15] and it might be related to the lower specificity of ATX compared with the gold standard. Therefore, strengthening the laboratory test of patients' related diseases and reducing the interference of related diseases or problems on serum ATX test might be able to enhance the specificity of ATX in PC patients. LPA has better sensitivity but lower specificity, particularly for detection of late stage disease.

Detection of disease based on a single tumor biomarker can have certain limitations, whereas combined detection of multiple indicators can yield higher sensitivity and specificity, and thus would have important clinical value for the early diagnosis of cancers such as PC [29]. In this study, we found that the AUROC for LPA alone or in combination with ATX or CA19-9 could significantly enhance its sensitivity for detection of early PC (TS1 and 2) relative to HVs and BPD patients, but this increase was not preserved for late stage (TS3 and 4) PC. Taken together, a combination of ATX, LPA, and CA19-9 as serum biomarkers may be a promising approach for early diagnosis of PC.

This study has several potential limitations. First, all data were compiled from patients treated at a single center. Inclusion of more diverse samples acquired from multiple centers is needed to verify the results of this study. Second, other biomarkers that have been used for early diagnosis of PC, such as CA125, CA242 and CA50, were not considered in this study. Third, this study did not include patients having other periampullary tumors such as distal cholangiocarcinomas, pancreatic neuroendocrine tumors and ampullary/duodenal cancers. Last, a large-scale study to examine tumor biomarker combinations and evaluate strategies for early diagnosis of PC is needed.

## Conclusion

This study performed a quantitative survey of the diagnostic potential of three biomarkers ATX, LPA and CA19-9 for the early detection of PC. Compared with HVs, the serum levels of all three biomarkers increased substantially in samples from patients with early stage PC. Moreover, the AUROC and diagnostic efficiency was enhanced significantly by the addition of ATX and LPA to CA19-9. These results showed that combined application of LPA and ATX with CA19-9 can be used for early detection of PC.

## Data Availability

The data that support the findings of this study are available from the general hospital of northern theater command but restrictions apply to the availability of these data, which were used under license for the current study, and so are not publicly available. Data are however available from the authors upon reasonable request and with permission of the general hospital of northern theater command.

## Abbreviations

PC: Pancreatic cancer
ATX: Autotaxin
LPA: Lysophosphatidic acidhealthy volunteers (HVs
BPDs: Benign pancreatic diseaseslysophospholipase D (lysoPLD)-
LPC: Lysophosphatidylcholine
ENPP2: Ectonucleotide pyrophosphatase/ phosphodiesterase 2receiver
ROC: Operating characteristics
AUROC: Areas Under the ROC Curve
